# Supplementary figures and images for: Inverted translational control of eukaryotic gene expression by ribosome collisions
Source: PLoS Biol. 2019 Sep 18;17(9):e3000396. doi: 10.1371/journal.pbio.3000396 (PMC6750593; doi:10.1371/journal.pbio.3000396)

**A**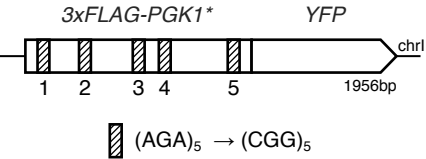**B**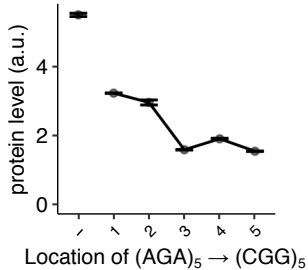**C**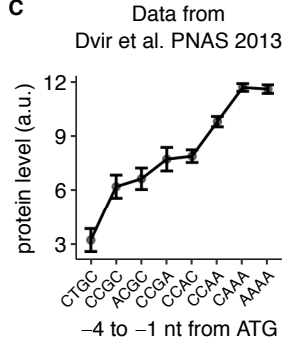

Supplement: S1 Fig — (A) Schematic of 3×FLAG-PGK1*-YFP reporters used in (B). The hatched regions indicate the five locations where an (AGA)5 is inserted into PGK1. One of these locations is synonymously mutated to (CGG)5 in the constructs shown in (B) (along with the no-mutation control). (B) Protein levels of 3×FLAG-PGK1*-YFP reporters with (CGG)5 inserted at one of the five locations indicated in (A). The no-mutation control is shown as −. Protein levels are quantified as the mean fluorescence of 10,000 cells for each strain using flow cytometry. Error bars show standard error of the mean over 4 independent yeast transformants. Protein levels are expressed as a.u. relative to the mean RFP levels from a constitutively expressed mKate2 control. (C) Protein levels of YFP library with randomized 10-nucleotide region preceding the ATG start codon. Data are from Dvir and colleagues [38]. Measured protein levels of all constructs with the same –4 to –1 nucleotides preceding ATG are averaged, and the error bars represent standard error of this average. The underlying data for panels B and C can be found at https://github.com/rasilab/ribosome_collisions_yeast. a.u., arbitrary unit; RFP, red fluorescent protein; YFP, yellow fluorescent protein. (PDF) [file pbio.3000396.s001.pdf]

**A**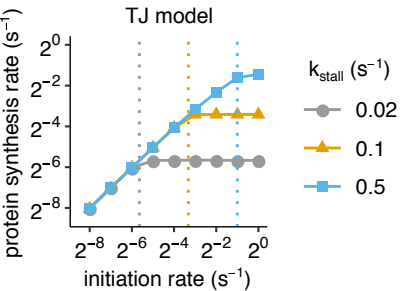**B**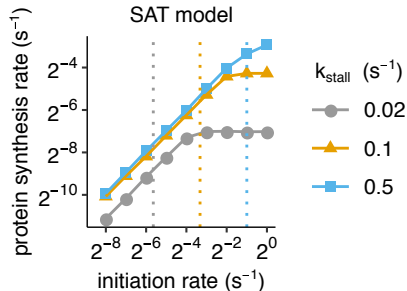**C**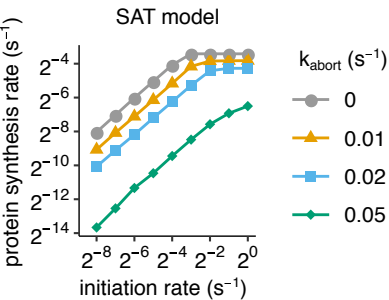**D**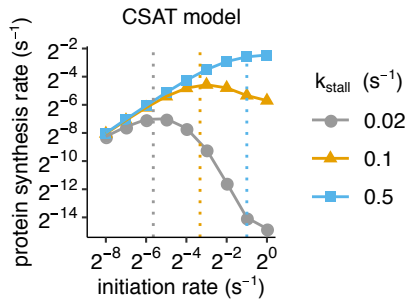

Supplement: S2 Fig — (A, B, D) Protein synthesis rate as a function of initiation rate for different rates of elongation at the ribosome stall in the TJ (A), SAT (B), and CSAT (D) models. The different elongation rates at the stall are indicated graphically as vertical dashed lines for comparison with initiation rate. (C) Protein synthesis rate as a function of initiation rate for different rates of abortive termination at the ribosome stall in the SAT model. The mRNA is 650 codons long, and the stall is encoded by six slowly translated codons located after 400 codons from the start. All other model parameters are listed in S3 Table. The underlying data for panels A, B, C, and D can be found at https://github.com/rasilab/ribosome_collisions_yeast. CSAT, collision-stimulated abortive termination; SAT, simple abortive termination; TJ, traffic jam. (PDF) [file pbio.3000396.s002.pdf]

**A**

SEC model

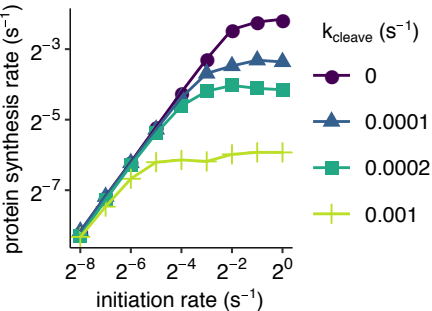**B**

CSEC model

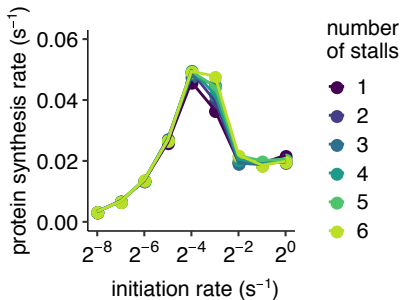

Supplement: S3 Fig — (A) Protein synthesis rate as a function of initiation rate for different rates of cotranslational endonucleolytic cleavage in the SEC model. (B) Protein synthesis rate as a function of initiation rate for different number of codons encoding the ribosome stall in the CSEC model. The mRNA is 650 codons long, and the stall is encoded by six slowly translated codons located after 400 codons from the start in (A). All other model parameters are listed in S3 Table. The underlying data for panels A and B can be found at https://github.com/rasilab/ribosome_collisions_yeast. CSEC, collision-stimulated endonucleolytic cleavage; SEC, simple endonucleolytic cleavage. (PDF) [file pbio.3000396.s003.pdf]

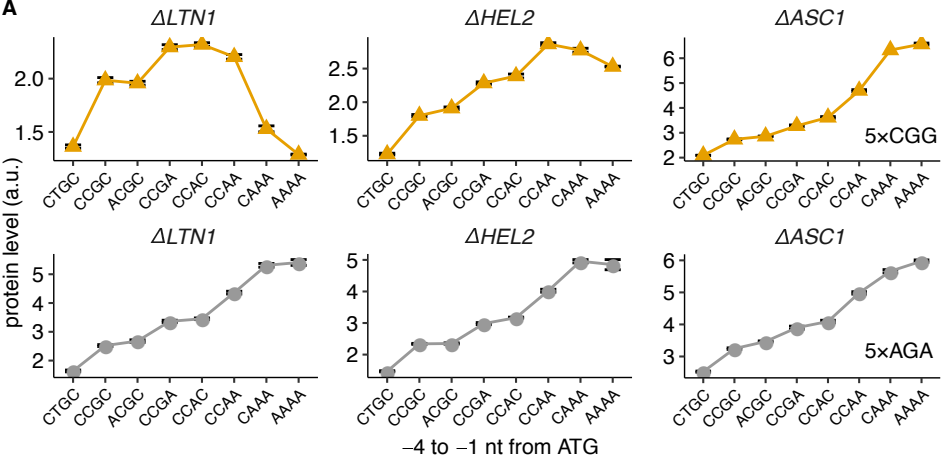

**B**

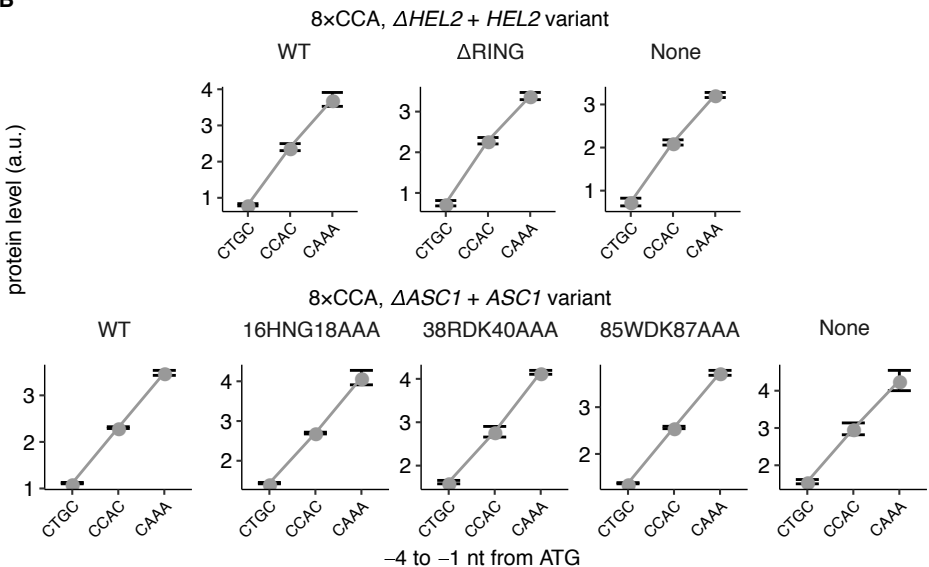

**C**

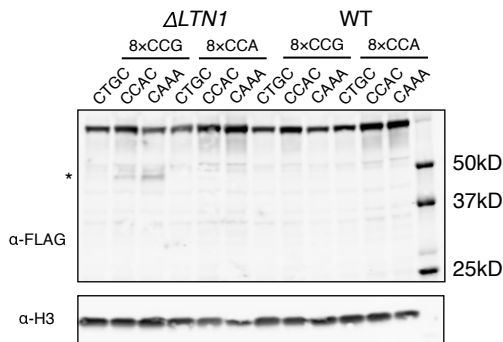

Supplement: S4 Fig — (A) Protein levels of 3×FLAG-PGK1*-YFP reporters (see Fig 1A) with varying initiation rates and with stall (5×CGG) or control (5×AGA) repeats. The reporters were integrated into the genome of isogenic strains with individual full deletions of LTN1, HEL2, or ASC1. (B) Protein levels of the 8×CCA control reporter expressed in either ΔHEL2 (top) or ΔASC1 (bottom) strain and complemented with the indicated HEL2 or ASC1 variant, respectively. (C) Western blots of 8×CCG and 8×CCA reporters with varying initiation rates and expressed in either ΔLTN1 or WT strain. Antibody against the FLAG epitope at the N terminus was used for detecting both full-length 3×FLAG-PGK1*-YFP and the truncated 3×FLAG-PGK1* due to abortive termination at the 8×CCG stall. * indicates the expected size (45 kD) of the truncated peptide. Histone H3 level is shown as loading control. Error bars in (A) and (B) show standard error over 3 or 4 independent transformants. The ΔASC1-CAAA-5×CGG variant alone has a single transformant. The underlying data for panels A and B can be found at https://github.com/rasilab/ribosome_collisions_yeast. WT, wild-type; YFP, yellow fluorescent protein. (PDF) [file pbio.3000396.s004.pdf]

**A**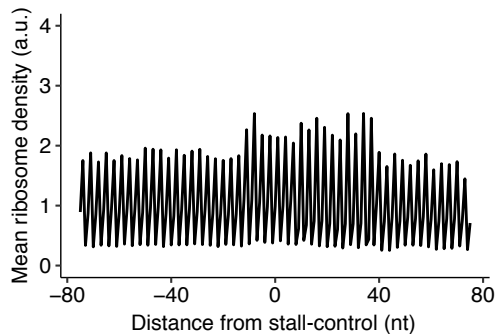**B**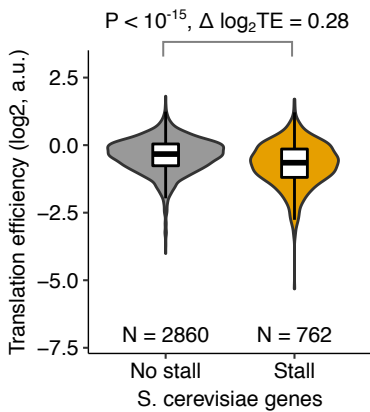

Supplement: S5 Fig — (A) Mean ribosome density around control sequences using data from Weinberg and colleagues [62]. Control sequences are defined as 10-codon windows that have a minimum of 6 glutamate or aspartate codons. A total of 1,552 S. cerevisiae mRNAs have at least one such control sequence. The ribosome density is normalized within the window around each control sequence before calculating the mean across all sequences. (B) TE of S. cerevisiae mRNA regions 5′ to stall sequences. TE is defined as the normalized ratio of ribosome footprint counts to total mRNA counts of regions 5′ to stalls for stall-containing mRNAs or the region on control mRNAs 5′ to the median stall location (215 codons) on stall-containing mRNAs. The underlying data for panels A and B can be found at https://github.com/rasilab/ribosome_collisions_yeast. TE, translation efficiency. (PDF) [file pbio.3000396.s005.pdf]
