# Supplementary material for: Inverted translational control of eukaryotic gene expression by ribosome collisions
Source: PLoS Biol. 2019 Sep 18;17(9):e3000396. doi: 10.1371/journal.pbio.3000396 (PMC6750593; doi:10.1371/journal.pbio.3000396)
Supplement: S1 Table — (PDF) [file pbio.3000396.s006.pdf]

Table S1. List of plasmids used for this study

Plasmid	Genotype	Figure	Source + Comment
p41894	chrI-URA3-pGPD-ccdB-Cyc1t	parent	Addgene 41894, single copy integration vector for ChrI safe harbor locus with URA3 selection marker
pHPSC16	HO3-LEU2-pGPD-mKate2-Cyc1t	parent	This work, single copy integration vector for HO locus with LEU2 selection marker
pSB2273	HIS3-pGPD-osT-Adh1t	parent	Gift from Sue Biggins lab, single copy integration vector for HIS3 locus with HIS3 selection marker
pHPSC417	p41894-CAAA-ATG-3xFLAG-PGK1-YFP	1,6	This work, exp133
pHPSC418	p41894-CCGC-ATG-3xFLAG-PGK1-YFP	1	This work, exp133
pHPSC419	p41894-CCAA-ATG-3xFLAG-PGK1-YFP	1	This work, exp133
pHPSC420	p41894-CCAC-ATG-3xFLAG-PGK1-YFP	1	This work, exp133
pHPSC421	p41894-CCGA-ATG-3xFLAG-PGK1-YFP	1	This work, exp133
pHPSC422	p41894-CTGC-ATG-3xFLAG-PGK1-YFP	1	This work, exp133
pHPSC423	p41894-AAAA-ATG-3xFLAG-PGK1-YFP	1	This work, exp133
pHPSC424	p41894-ACGC-ATG-3xFLAG-PGK1-YFP	1	This work, exp133
pHPSC425	p41894-CAAA-CTG-3xFLAG-PGK1-YFP	1	This work, exp133
pHPSC354	p41894-CAAA-ATG-3xFLAG-PGK1*-10xAAG-YFP	1	This work, exp98
pHPSC355	p41894-CCGC-ATG-3xFLAG-PGK1*-10xAAG-YFP	1	This work, exp98
pHPSC356	p41894-CCAA-ATG-3xFLAG-PGK1*-10xAAG-YFP	1	This work, exp98
pHPSC357	p41894-CCAC-ATG-3xFLAG-PGK1*-10xAAG-YFP	1	This work, exp98
pHPSC358	p41894-CCGA-ATG-3xFLAG-PGK1*-10xAAG-YFP	1	This work, exp98
pHPSC359	p41894-CTGC-ATG-3xFLAG-PGK1*-10xAAG-YFP	1	This work, exp98
pHPSC360	p41894-AAAA-ATG-3xFLAG-PGK1*-10xAAG-YFP	1	This work, exp98
pHPSC361	p41894-ACGC-ATG-3xFLAG-PGK1*-10xAAG-YFP	1	This work, exp98
pHPSC363	p41894-CAAA-ATG-3xFLAG-PGK1*-10xAGA-YFP	1	This work, exp98
pHPSC364	p41894-CCGC-ATG-3xFLAG-PGK1*-10xAGA-YFP	1	This work, exp98
pHPSC365	p41894-CCAA-ATG-3xFLAG-PGK1*-10xAGA-YFP	1	This work, exp98
pHPSC366	p41894-CCAC-ATG-3xFLAG-PGK1*-10xAGA-YFP	1	This work, exp98
pHPSC367	p41894-CCGA-ATG-3xFLAG-PGK1*-10xAGA-YFP	1	This work, exp98
pHPSC368	p41894-CTGC-ATG-3xFLAG-PGK1*-10xAGA-YFP	1	This work, exp98
pHPSC369	p41894-AAAA-ATG-3xFLAG-PGK1*-10xAGA-YFP	1	This work, exp98
pHPSC370	p41894-ACGC-ATG-3xFLAG-PGK1*-10xAGA-YFP	1	This work, exp98
pHPSC314	p41894-CAAA-ATG-3xFLAG-PGK1*-8xCCA-YFP	1	This work, exp93
pHPSC315	p41894-CCGC-ATG-3xFLAG-PGK1*-8xCCA-YFP	1	This work, exp93
pHPSC316	p41894-CCAA-ATG-3xFLAG-PGK1*-8xCCA-YFP	1	This work, exp93
pHPSC317	p41894-CCAC-ATG-3xFLAG-PGK1*-8xCCA-YFP	1	This work, exp93
pHPSC318	p41894-CCGA-ATG-3xFLAG-PGK1*-8xCCA-YFP	1	This work, exp93

Continued on next page

Continued from previous page			
**Plasmid**	**Genotype**	**Figure**	**Source + Comment**
pHPSC319	p41894-CTGC-ATG-3xFLAG-PGK1*-8xCCA-YFP	1	This work, exp93
pHPSC320	p41894-AAAA-ATG-3xFLAG-PGK1*-8xCCA-YFP	1	This work, exp93
pHPSC321	p41894-ACGC-ATG-3xFLAG-PGK1*-8xCCA-YFP	1	This work, exp93
pHPSC323	p41894-CAAA-ATG-3xFLAG-PGK1*-8xCCG-YFP	1	This work, exp93
pHPSC324	p41894-CCGC-ATG-3xFLAG-PGK1*-8xCCG-YFP	1	This work, exp93
pHPSC325	p41894-CCAA-ATG-3xFLAG-PGK1*-8xCCG-YFP	1	This work, exp93
pHPSC326	p41894-CCAC-ATG-3xFLAG-PGK1*-8xCCG-YFP	1	This work, exp93
pHPSC327	p41894-CCGA-ATG-3xFLAG-PGK1*-8xCCG-YFP	1	This work, exp93
pHPSC328	p41894-CTGC-ATG-3xFLAG-PGK1*-8xCCG-YFP	1	This work, exp93
pHPSC329	p41894-AAAA-ATG-3xFLAG-PGK1*-8xCCG-YFP	1	This work, exp93
pHPSC330	p41894-ACGC-ATG-3xFLAG-PGK1*-8xCCG-YFP	1	This work, exp93
pHPSC57	p41894-CAAA-ATG-3xFLAG-PGK1*-5xAGA-YFP	1	This work, exp60
pHPSC126	p41894-CCGC-ATG-3xFLAG-PGK1*-5xAGA-YFP	1	This work, exp60
pHPSC158	p41894-CCAA-ATG-3xFLAG-PGK1*-5xAGA-YFP	1	This work, exp60
pHPSC159	p41894-CCAC-ATG-3xFLAG-PGK1*-5xAGA-YFP	1	This work, exp60
pHPSC160	p41894-CCGA-ATG-3xFLAG-PGK1*-5xAGA-YFP	1	This work, exp60
pHPSC161	p41894-CTGC-ATG-3xFLAG-PGK1*-5xAGA-YFP	1	This work, exp60
pHPSC162	p41894-AAAA-ATG-3xFLAG-PGK1*-5xAGA-YFP	1	This work, exp60
pHPSC163	p41894-ACGC-ATG-3xFLAG-PGK1*-5xAGA-YFP	1	This work, exp60
pHPSC72	p41894-CAAA-ATG-3xFLAG-PGK1*-5xCGG-YFP	1	This work, exp60
pHPSC131	p41894-CCGC-ATG-3xFLAG-PGK1*-5xCGG-YFP	1	This work, exp60
pHPSC188	p41894-CCAA-ATG-3xFLAG-PGK1*-5xCGG-YFP	1	This work, exp60
pHPSC193	p41894-CCAC-ATG-3xFLAG-PGK1*-5xCGG-YFP	1	This work, exp60
pHPSC168	p41894-CCGA-ATG-3xFLAG-PGK1*-5xCGG-YFP	1	This work, exp60
pHPSC198	p41894-CTGC-ATG-3xFLAG-PGK1*-5xCGG-YFP	1	This work, exp60
pHPSC203	p41894-AAAA-ATG-3xFLAG-PGK1*-5xCGG-YFP	1	This work, exp60
pHPSC208	p41894-ACGC-ATG-3xFLAG-PGK1*-5xCGG-YFP	1	This work, exp60
pHPSC513	pSB2273-HEL2	5	This work, exp153
pHPSC515	pSB2273-HEL2-delring	5	This work, exp153
pHPSC516	pSB2273-ASC1	5	This work, exp153
pHPSC517	pSB2273-ASC1-h16	5	This work, exp153
pHPSC518	pSB2273-ASC1-r38	5	This work, exp153
pHPSC519	pSB2273-ASC1-w85	5	This work, exp153
pHPSC512	Barcoded plasmid pool (see plasmid cloning methods)	2,5	This work, exp152
pHPSC758	p41894-PGK1-MAP2stall-YFP	6	This work, exp176
pHPSC759	p41894-PGK1-PAR32stall-YFP	6	This work, exp176
pHPSC760	p41894-PGK1-HMO1stall-YFP	6	This work, exp176
pHPSC761	p41894-PGK1-RMP1stall-YFP	6	This work, exp176
pHPSC762	p41894-PGK1-TMA23stall-YFP	6	This work, exp176
pHPSC763	p41894-PGK1-JJJ1stall-YFP	6	This work, exp176
