# Supplementary material for: Inverted translational control of eukaryotic gene expression by ribosome collisions
Source: PLoS Biol. 2019 Sep 18;17(9):e3000396. doi: 10.1371/journal.pbio.3000396 (PMC6750593; doi:10.1371/journal.pbio.3000396)
Supplement: S2 Table — (PDF) [file pbio.3000396.s007.pdf]

Table S2. List of *S. cerevisiae* strains used in this study

Strain	Genotype, integrated plasmid	Figure	Source + Comment
BY4741	S288C, MATa HIS3$Δ$1 LEU2$Δ$0 MET15$Δ$0 URA3$Δ$0	Parent	Thermo Fisher
scHP15	BY4741, pHPSC16	Parent	This work
scAS12	scHP15, LTN1::KAN	Parent	This work
scAS17	scHP15, DOM34::KAN	Parent	This work
scHP498	scHP15, ASC1::NAT	Parent	This work
scHP520	scHP15, HEL2::NAT	Parent	This work
scHP1125	scHP15, pHPSC512	2	This work, exp152
scHP1126	scAS12, pHPSC512	5	This work, exp152
scHP1127	scAS17, pHPSC512	5	This work, exp152
scHP1128	scHP498, pHPSC512	5	This work, exp152
scHP1129	scHP520, pHPSC512	5	This work, exp152
scHP971	scHP15, pHPSC417	1	This work, exp133
scHP972	scHP15, pHPSC418	1	This work, exp133
scHP973	scHP15, pHPSC419	1	This work, exp133
scHP974	scHP15, pHPSC420	1	This work, exp133
scHP975	scHP15, pHPSC421	1	This work, exp133
scHP976	scHP15, pHPSC422	1	This work, exp133
scHP977	scHP15, pHPSC423	1	This work, exp133
scHP978	scHP15, pHPSC424	1	This work, exp133
scHP674	scHP15, pHPSC354	1	This work, exp98
scHP675	scHP15, pHPSC355	1	This work, exp98
scHP676	scHP15, pHPSC356	1	This work, exp98
scHP677	scHP15, pHPSC357	1	This work, exp98
scHP678	scHP15, pHPSC358	1	This work, exp98
scHP679	scHP15, pHPSC359	1	This work, exp98
scHP680	scHP15, pHPSC360	1	This work, exp98
scHP681	scHP15, pHPSC361	1	This work, exp98
scHP683	scHP15, pHPSC363	1	This work, exp98
scHP684	scHP15, pHPSC364	1	This work, exp98
scHP685	scHP15, pHPSC365	1	This work, exp98
scHP686	scHP15, pHPSC366	1	This work, exp98
scHP687	scHP15, pHPSC367	1	This work, exp98
scHP688	scHP15, pHPSC368	1	This work, exp98
scHP689	scHP15, pHPSC369	1	This work, exp98
scHP690	scHP15, pHPSC370	1	This work, exp98
			,
scHP617	scHP15, pHPSC314	1	This work, exp93
scHP618	scHP15, pHPSC315	1	This work, exp93
scHP619	scHP15, pHPSC316	1	This work, exp93
scHP620	scHP15, pHPSC317	1	This work, exp93
scHP621	scHP15, pHPSC318	1	This work, exp93
scHP622	scHP15, pHPSC319	1	This work, exp93
scHP623	scHP15, pHPSC320	1	This work, exp93
scHP624	scHP15, pHPSC321	1	This work, exp93
scHP626	scHP15, pHPSC323	1	This work, exp93
scHP627	scHP15, pHPSC324	1	This work, exp93

Continued on next page

Continued from previous page

**Strain**	**Genotype, integrated plasmid**	**Figure**	**Source + Comment**
scHP628	scHP15, pHPSC325	1	This work, exp93
scHP629	scHP15, pHPSC326	1	This work, exp93
scHP630	scHP15, pHPSC327	1	This work, exp93
scHP631	scHP15, pHPSC328	1	This work, exp93
scHP632	scHP15, pHPSC329	1	This work, exp93
scHP633	scHP15, pHPSC330	1	This work, exp93
scHP76	scHP15, pHPSC57	1	This work, exp60
scHP310	scHP15, pHPSC126	1	This work, exp60
scHP311	scHP15, pHPSC158	1	This work, exp60
scHP312	scHP15, pHPSC159	1	This work, exp60
scHP313	scHP15, pHPSC160	1	This work, exp60
scHP314	scHP15, pHPSC161	1	This work, exp60
scHP315	scHP15, pHPSC162	1	This work, exp60
scHP316	scHP15, pHPSC163	1	This work, exp60
scHP91	scHP15, pHPSC72	1	This work, exp60
scHP271	scHP15, pHPSC131	1	This work, exp60
scHP276	scHP15, pHPSC188	1	This work, exp60
scHP281	scHP15, pHPSC193	1	This work, exp60
scHP266	scHP15, pHPSC168	1	This work, exp60
scHP286	scHP15, pHPSC198	1	This work, exp60
scHP291	scHP15, pHPSC203	1	This work, exp60
scHP296	scHP15, pHPSC208	1	This work, exp60
scHP747	scHP15, pHPSC314	5	This work, exp114
scHP748	scHP15, pHPSC317	5	This work, exp114
scHP749	scHP15, pHPSC319	5	This work, exp114
scHP750	scHP15, pHPSC323	5	This work, exp114
scHP751	scHP15, pHPSC326	5	This work, exp114
scHP752	scHP15, pHPSC328	5	This work, exp114
scHP759	scAS12, pHPSC314	5	This work, exp114
scHP760	scAS12, pHPSC317	5	This work, exp114
scHP761	scAS12, pHPSC319	5	This work, exp114
scHP762	scAS12, pHPSC323	5	This work, exp114
scHP763	scAS12, pHPSC326	5	This work, exp114
scHP764	scAS12, pHPSC328	5	This work, exp114
scHP765	scAS17, pHPSC314	5	This work, exp114
scHP766	scAS17, pHPSC317	5	This work, exp114
scHP767	scAS17, pHPSC319	5	This work, exp114
scHP768	scAS17, pHPSC323	5	This work, exp114
scHP769	scAS17, pHPSC326	5	This work, exp114
scHP770	scAS17, pHPSC328	5	This work, exp114
scHP771	scHP498, pHPSC314	5	This work, exp114
scHP772	scHP498, pHPSC317	5	This work, exp114
scHP773	scHP498, pHPSC319	5	This work, exp114
scHP774	scHP498, pHPSC323	5	This work, exp114
scHP775	scHP498, pHPSC326	5	This work, exp114

Continued on next page

Continued from previous page

Strain	Genotype, integrated plasmid	Figure	Source + Comment
scHP776	scHP498, pHPSC328	5	This work, exp114
scHP777	scHP520, pHPSC314	5	This work, exp114
scHP778	scHP520, pHPSC317	5	This work, exp114
scHP779	scHP520, pHPSC319	5	This work, exp114
scHP780	scHP520, pHPSC323	5	This work, exp114
scHP781	scHP520, pHPSC326	5	This work, exp114
scHP782	scHP520, pHPSC328	5	This work, exp114
scHP521	scAS12, pHPSC57	S4	This work, exp70
scHP531	scAS12, pHPSC126	S4	This work, exp70
scHP532	scAS12, pHPSC158	S4	This work, exp70
scHP533	scAS12, pHPSC159	S4	This work, exp70
scHP534	scAS12, pHPSC160	S4	This work, exp70
scHP535	scAS12, pHPSC161	S4	This work, exp70
scHP536	scAS12, pHPSC162	S4	This work, exp70
scHP537	scAS12, pHPSC163	S4	This work, exp70
scHP522	scAS12, pHPSC72	S4	This work, exp70
scHP523	scAS12, pHPSC168	S4	This work, exp70
scHP524	scAS12, pHPSC131	S4	This work, exp70
scHP525	scAS12, pHPSC188	S4	This work, exp70
scHP526	scAS12, pHPSC193	S4	This work, exp70
scHP527	scAS12, pHPSC198	S4	This work, exp70
scHP528	scAS12, pHPSC203	S4	This work, exp70
scHP529	scAS12, pHPSC208	S4	This work, exp70
scHP539	scHP498, pHPSC57	S4	This work, exp70
scHP549	scHP498, pHPSC126	S4	This work, exp70
scHP550	scHP498, pHPSC158	S4	This work, exp70
scHP551	scHP498, pHPSC159	S4	This work, exp70
scHP552	scHP498, pHPSC160	S4	This work, exp70
scHP553	scHP498, pHPSC161	S4	This work, exp70
scHP554	scHP498, pHPSC162	S4	This work, exp70
scHP555	scHP498, pHPSC163	S4	This work, exp70
scHP540	scHP498, pHPSC72	S4	This work, exp70
scHP541	scHP498, pHPSC168	S4	This work, exp70
scHP542	scHP498, pHPSC131	S4	This work, exp70
scHP543	scHP498, pHPSC188	S4	This work, exp70
scHP544	scHP498, pHPSC193	S4	This work, exp70
scHP545	scHP498, pHPSC198	S4	This work, exp70
scHP546	scHP498, pHPSC203	S4	This work, exp70
scHP547	scHP498, pHPSC208	S4	This work, exp70
scHP557	scHP520, pHPSC57	S4	This work, exp70
scHP567	scHP520, pHPSC126	S4	This work, exp70
scHP568	scHP520, pHPSC158	S4	This work, exp70
scHP569	scHP520, pHPSC159	S4	This work, exp70
scHP570	scHP520, pHPSC160	S4	This work, exp70
scHP571	scHP520, pHPSC161	S4	This work, exp70
scHP572	scHP520, pHPSC162	S4	This work, exp70

Continued on next page

Continued from previous page

Strain	Genotype, integrated plasmid	Figure	Source + Comment
scHP573	scHP520, pHPSC163	S4	This work, exp70
scHP558	scHP520, pHPSC72	S4	This work, exp70
scHP559	scHP520, pHPSC168	S4	This work, exp70
scHP560	scHP520, pHPSC131	S4	This work, exp70
scHP561	scHP520, pHPSC188	S4	This work, exp70
scHP562	scHP520, pHPSC193	S4	This work, exp70
scHP563	scHP520, pHPSC198	S4	This work, exp70
scHP564	scHP520, pHPSC203	S4	This work, exp70
scHP565	scHP520, pHPSC208	S4	This work, exp70
scHP1130	scHP520, pHPSC513	5	This work, exp153
scHP1132	scHP520, pHPSC515	5	This work, exp153
scHP1133	scHP498, pHPSC516	5	This work, exp153
scHP1134	scHP498, pHPSC517	5	This work, exp153
scHP1135	scHP498, pHPSC518	5	This work, exp153
scHP1136	scHP498, pHPSC519	5	This work, exp153
scHP1137	scHP1130, pHPSC314	5	This work, exp153
scHP1138	scHP1130, pHPSC317	5	This work, exp153
scHP1139	scHP1130, pHPSC319	5	This work, exp153
scHP1140	scHP1130, pHPSC323	5	This work, exp153
scHP1141	scHP1130, pHPSC326	5	This work, exp153
scHP1142	scHP1130, pHPSC328	5	This work, exp153
scHP1143	scHP1131, pHPSC314	5	This work, exp153
scHP1144	scHP1131, pHPSC317	5	This work, exp153
scHP1145	scHP1131, pHPSC319	5	This work, exp153
scHP1146	scHP1131, pHPSC323	5	This work, exp153
scHP1147	scHP1131, pHPSC326	5	This work, exp153
scHP1148	scHP1131, pHPSC328	5	This work, exp153
scHP1149	scHP1132, pHPSC314	5	This work, exp153
scHP1150	scHP1132, pHPSC317	5	This work, exp153
scHP1151	scHP1132, pHPSC319	5	This work, exp153
scHP1152	scHP1132, pHPSC323	5	This work, exp153
scHP1153	scHP1132, pHPSC326	5	This work, exp153
scHP1154	scHP1132, pHPSC328	5	This work, exp153
scHP1155	scHP1133, pHPSC314	5	This work, exp153
scHP1156	scHP1133, pHPSC317	5	This work, exp153
scHP1157	scHP1133, pHPSC319	5	This work, exp153
scHP1158	scHP1133, pHPSC323	5	This work, exp153
scHP1159	scHP1133, pHPSC326	5	This work, exp153
scHP1160	scHP1133, pHPSC328	5	This work, exp153
scHP1161	scHP1134, pHPSC314	5	This work, exp153
scHP1162	scHP1134, pHPSC317	5	This work, exp153
scHP1163	scHP1134, pHPSC319	5	This work, exp153
scHP1164	scHP1134, pHPSC323	5	This work, exp153
scHP1165	scHP1134, pHPSC326	5	This work, exp153

Continued on next page

Continued from previous page			
**Strain**	**Genotype, integrated plasmid**	**Figure**	**Source + Comment**
scHP1166	scHP1134, pHPSC328	5	This work, exp153
scHP1167	scHP1135, pHPSC314	5	This work, exp153
scHP1168	scHP1135, pHPSC317	5	This work, exp153
scHP1169	scHP1135, pHPSC319	5	This work, exp153
scHP1170	scHP1135, pHPSC323	5	This work, exp153
scHP1171	scHP1135, pHPSC326	5	This work, exp153
scHP1172	scHP1135, pHPSC328	5	This work, exp153
scHP1173	scHP1136, pHPSC314	5	This work, exp153
scHP1174	scHP1136, pHPSC317	5	This work, exp153
scHP1175	scHP1136, pHPSC319	5	This work, exp153
scHP1176	scHP1136, pHPSC323	5	This work, exp153
scHP1177	scHP1136, pHPSC326	5	This work, exp153
scHP1178	scHP1136, pHPSC328	5	This work, exp153
scHP1490	scHP15, pHPSC758	6	This work, exp176
scHP1492	scHP15, pHPSC759	6	This work, exp176
scHP1493	scHP15, pHPSC760	6	This work, exp176
scHP1494	scHP15, pHPSC761	6	This work, exp176
scHP1495	scHP15, pHPSC762	6	This work, exp176
scHP1496	scHP15, pHPSC763	6	This work, exp176
scHP1497	scHP15, pHPSC417	6	This work, exp176
scHP1498	scHP498, pHPSC758	6	This work, exp176
scHP1500	scHP498, pHPSC759	6	This work, exp176
scHP1501	scHP498, pHPSC760	6	This work, exp176
scHP1502	scHP498, pHPSC761	6	This work, exp176
scHP1503	scHP498, pHPSC762	6	This work, exp176
scHP1504	scHP498, pHPSC763	6	This work, exp176
scHP1505	scHP498, pHPSC417	6	This work, exp176
