# Supplementary material for: Inverted translational control of eukaryotic gene expression by ribosome collisions
Source: PLoS Biol. 2019 Sep 18;17(9):e3000396. doi: 10.1371/journal.pbio.3000396 (PMC6750593; doi:10.1371/journal.pbio.3000396)
Supplement: S3 Table — (PDF) [file pbio.3000396.s008.pdf]

Table S3. Simulation Parameters. Default parameter values across all simulations are listed first. Parameters altered for specific simulations are listed next under the corresponding figure. Parameters not listed for a specific figure are either set to the default value or explicitly indicated to be the same as for another figure. N.U. stands for No Units. The symbol names correspond to the ones used in `tasep.py`. For parameters that are not systematically varied in our work but that have been previously measured, we chose them to be a convenient rounded value within 2-fold of the measured value. For example, we set the ribosome footprint size to 10 codons while it is closer to 9 codons (Ingolia et al. 2009); The decapping rate of *PGK1* mRNA is set to $0.01 s^{-1}$ while it is measured to be $0.008 s^{-1}$ (Cao et al. 2001). We do not expect these choices to alter any of the results presented in this study.

Symbol	Value	Unit	Comment
**Default**			
l_{ribo}	10	*codon*	Ribosome fooprint size on *S. cerevisiae* mRNAs (Ingolia et al. 2009).
l_{mrna}	650	*codon*	Approximate size of *3xFLAG-PGK1*-YFP* reporters used in our experiments.
k_{init}	1	s^{-1}	Initiation rate, Systematically varied in our work.
k_{elong}	10	$codon/s^{-1}$	Normal elongation rate on *S. cerevisiae* mRNAs (Boehlke et al. 1975).
k_{term}	1	s^{-1}	Normal termination rate, set lower than k_{elong} but not lower than k_{init}. Exact value is arbitrary and does not affect our prediction.
$k_{transcription}$	0.001	s^{-1}	Transcription rate of reporter mRNA. Exact value is arbitrary and does not affect our prediction.
l_{polya}	60	*nt*	Length of poly-A tail, similar to (Cao et al. 2001). Systematically varied in our work.
$k_{deadenylation}$	0.03	nt/s^{-1}	Deadenylation, based on (Cao et al. 2001). Exact value sets the lifetime of mRNAs degraded through the canonical decay pathway.
$k_{decapping}$	0.01	s^{-1}	Decapping, based on (Cao et al. 2001). Exact value sets the lifetime of mRNAs degraded through the canonical decay pathway.
k_{exo_53}	1.0	$codon/s^{-1}$	3× larger than (Cao et al. 2001). Exact value is arbitrary and set higher than $k_{deadenylation}$ and $k_{decapping}$.
$k_{elong\_stall\_1}$	0.1	$codon/s^{-1}$	Elongation rate at the single stall codon located at x_{stall_1}. Systematically varied in our work.
x_{stall_1}	400	*codon*	Location of stall. Approximate location of stalls in the *3xFLAG-PGK1*-YFP* reporters used in our experiments.
n_{stall}	1	N.U.	Number of stall codons. Systematically varied in our work.
$k_{preterm\_no\_hit\_intact}$	0.01	s^{-1}	Abortive termination rate of ribosomes on intact mRNAs that are not hit from either mRNA entry or exit side. Systematically varied in our work.
$k_{preterm\_5\_hit\_intact}$	0.01	s^{-1}	Abortive termination rate of ribosomes on intact mRNAs that are hit from mRNA exit side but not entry side. Systematically varied in our work.
$k_{preterm\_3\_hit\_intact}$	0.01	s^{-1}	Abortive termination rate of ribosomes on intact mRNAs that are hit from mRNA entry side but not exit side. Systematically varied in our work.

Continued on next page

Continued from previous page

Symbol	Value	Unit	Comment
$k_{preterm\_both\_hit\_intact}$	0.01	s^{-1}	Abortive termination rate of ribosomes on intact mRNAs that are hit from both mRNA entry and exit sides. Systematically varied in our work.
$k_{preterm\_no\_hit\_endocleaved}$	1.0	s^{-1}	Abortive termination rate of ribosomes with truncated mRNAs in A-site that are not hit from either mRNA entry or exit side. Systematically varied in our work.
$k_{preterm\_5\_hit\_endocleaved}$	1.0	s^{-1}	Abortive termination rate of ribosomes with truncated mRNAs in A-site that are hit from mRNA exit side but not entry side. Systematically varied in our work.
$k_{preterm\_3\_hit\_endocleaved}$	1.0	s^{-1}	Abortive termination rate of ribosomes with truncated mRNAs in A-site that are hit from mRNA entry side but not exit side. Systematically varied in our work.
$k_{preterm\_both\_hit\_endocleaved}$	1.0	s^{-1}	Abortive termination rate of ribosomes with truncated mRNAs in A-site that are hit from both mRNA entry and exit sides. Systematically varied in our work.
$k_{cleave\_no\_hit}$	0.0001	s^{-1}	Endonucleolytic mRNA cleavage rate at ribosomes that are not hit from either mRNA entry or exit side. Systematically varied in our work.
$k_{cleave\_5\_hit}$	0.0001	s^{-1}	Endonucleolytic mRNA cleavage rate at ribosomes that are high from mRNA exit side but not entry side. Systematically varied in our work.
$k_{cleave\_3\_hit}$	0.0001	s^{-1}	Endonucleolytic mRNA cleavage rate at ribosomes that are high from mRNA entry side but not exit side. Systematically varied in our work.
$k_{cleave\_both\_hit}$	0.0001	s^{-1}	Endonucleolytic mRNA cleavage rate at ribosomes that are hit from both mRNA entry and exit sides. Systematically varied in our work.
n_{dna_0}	1	N.U.	Initial copy number of our reporter gene for transcription. Exact value is arbitrary and does not affect our prediction.
n_{mrna_0}	0	N.U.	Initial copy number of our reporter mRNA. Exact value is arbitrary and does not affect our prediction.
$n_{ribosome\_0}$	10000	N.U.	Initial number of ribosomes in the simulation. Exact value is arbitrary and does not affect our prediction since we directly fix the initiation rate of mRNAs (k_{init}).
$n_{protein\_0}$	0	N.U.	Initial number of full proteins in the simulation.
$n_{abortedprotein\_0}$	0	N.U.	Initial number of aborted proteins in the simulation.
`-sim`	10^6	s	Biological time simulated, `NFsim` parameter.
`-seed`	111	N.U.	Seed for random draws during simulation, `NFsim` parameter.
`-utl`	3	N.U.	Maximum number of connected molecules examined for updates, `NFsim` parameter.
`-gml`	10^6	N.U.	Maximum number of molecules allowed in the simulation, `NFsim` parameter.
`-maxcputime`	6000	s	Maximum CPU time per simulation, `NFsim` parameter (present only in our customized version).
`-network`	—	—	Switch telling `NFsim` to infer reaction dependency at start and use it for updates (switch present only in our customized version).
**Fig. 3B**			**Common parameters for all models are listed first.**

Continued on next page

Continued from previous page

Symbol	Value	Unit	Comment
k_{init}	0.004 – 1	s^{-1}	Initiation rate, Varied in 2-fold increments.
$k_{transcription}$	0	s^{-1}	Transcription rate of reporter mRNA set to zero, single mRNA present throughout ($n_{mrna\_0} = 1$).
$k_{deadenylation}$	0	nt/s^{-1}	Decay rate of reporter mRNA set to zero, single mRNA present throughout.
n_{mrna_0}	1	N.U.	Initial copy number of our reporter mRNA set to 1, and remains unchanged throughout the simulation.
x_{stall}	401:406	*codon*	Six consecutive stall codons.
k_{elong_stall}	0.6	$codon/s^{-1}$	Total elongation rate across 6 stall codons set to $0.1s^{-1}$.
n_{stall}	6	N.U.	Number of stall codons.
$k_{cleave\_no\_hit}$, $k_{cleave\_5\_hit}$, $k_{cleave\_3\_hit}$, $k_{cleave\_both\_hit}$	0	s^{-1}	No endonucleolytic cleavage at ribosomes.
**Fig. 3B, TJ model**			
$k_{preterm\_no\_hit\_intact}$, $k_{preterm\_5\_hit\_intact}$, $k_{preterm\_3\_hit\_intact}$, $k_{preterm\_both\_hit\_intact}$	0	s^{-1}	No abortive termination.
**Fig. 3B, SAT model**			
$k_{preterm\_no\_hit\_intact}$, $k_{preterm\_5\_hit\_intact}$	0.02	s^{-1}	Ribosomes that are not hit or hit from mRNA exit side abort with the same rate.
$k_{preterm\_3\_hit\_intact}$, $k_{preterm\_both\_hit\_intact}$	0	s^{-1}	Ribosomes that are hit from both sides or only from the mRNA entry side do not abort.
**Fig. 3B, CSAT model**			
$k_{preterm\_5\_hit\_intact}$, $k_{preterm\_both\_hit\_intact}$	1	s^{-1}	Ribosomes that are hit only from mRNA exit side or both sides abort with the same rate.
$k_{preterm\_no\_hit\_intact}$, $k_{preterm\_3\_hit\_intact}$	0	s^{-1}	Ribosomes that are not hit or hit only from the mRNA entry side do not abort.
**Fig. 3B, CAT model**			
$k_{preterm\_3\_hit\_intact}$, $k_{preterm\_both\_hit\_intact}$	1	s^{-1}	Ribosomes that are hit only from mRNA entry side or both sides abort with the same rate.
$k_{preterm\_no\_hit\_intact}$, $k_{preterm\_5\_hit\_intact}$	0	s^{-1}	Ribosomes that are not hit or only from the mRNA exit side do not abort.
**Fig. 3C**			**All parameters except ones below same as CSAT model in Fig. 3B.**
**Fig. 3C**, $n_{stall} = 1$			
x_{stall}	401	*codon*	Location of stall codons.
k_{elong_stall}	0.1	$codon/s^{-1}$	Elongation rate per codon in increased as n_{stall} in increased to keep total elongation rate across stall constant.

Continued on next page

Continued from previous page

Symbol	Value	Unit	Comment
**Fig. 3C**, $n_{stall} = 2$			
x_{stall}	400:402	*codon*	Location of stall codons.
k_{elong_stall}	0.2	$codon/s^{-1}$	Elongation rate per codon in increased as n_{stall} in increased to keep total elongation rate across stall constant.
**Fig. 3C**, $n_{stall} = 3$			
x_{stall}	401:403	*codon*	Location of stall codons.
k_{elong_stall}	0.3	$codon/s^{-1}$	Elongation rate per codon in increased as n_{stall} in increased to keep total elongation rate across stall constant.
**Fig. 3C**, $n_{stall} = 4$			
x_{stall}	401:404	*codon*	Location of stall codons.
k_{elong_stall}	0.4	$codon/s^{-1}$	Elongation rate per codon in increased as n_{stall} in increased to keep total elongation rate across stall constant.
**Fig. 3C**, $n_{stall} = 5$			
x_{stall}	401:405	*codon*	Location of stall codons.
k_{elong_stall}	0.5	$codon/s^{-1}$	Elongation rate per codon in increased as n_{stall} in increased to keep total elongation rate across stall constant.
**Fig. 3C**, $n_{stall} = 6$			
x_{stall}	401:406	*codon*	Location of stall codons.
k_{elong_stall}	0.6	$codon/s^{-1}$	Elongation rate per codon in increased as n_{stall} in increased to keep total elongation rate across stall constant.
**S2 Fig panel A**			**All parameters except ones below same as TJ model in Fig. 3B.**
k_{elong_stall}	0.12, 0.6, 3.6	$codon/s^{-1}$	Total elongation rate across 6 stall codons set to $0.02, 1, 0.5s^{-1}$.
**S2 Fig panel B**			**All parameters except ones below same as SAT model in Fig. 3B.**
k_{elong_stall}	0.12, 0.6, 3.6	$codon/s^{-1}$	Total elongation rate across 6 stall codons set to $0.02, 1, 0.5s^{-1}$.
**S2 Fig panel C**			**All parameters except ones below same as SAT model in Fig. 3B.**
$k_{preterm\_no\_hit\_intact}$, $k_{preterm\_5\_hit\_intact}$	0, 0.01, 0.02, 0.05	s^{-1}	Abortive termination rate.
**S2 Fig panel D**			**All parameters except ones below same as CSAT model in Fig. 3B.**

Continued on next page

Continued from previous page

Symbol	Value	Unit	Comment
k_{elong_stall}	0.12, 0.6, 3.6	$codon/s^{-1}$	Total elongation rate across 6 stall codons set to $0.02, 1, 0.5s^{-1}$.
**Fig. 4B, Fig. 4C**			**Common parameters for all models are listed first.**
k_{init}	0.004 – 1	s^{-1}	Initiation rate, Varied in 2-fold increments.
x_{stall}	401:406	*codon*	Six consecutive stalling codons.
k_{elong_stall}	0.6	$codon/s^{-1}$	Total elongation rate across 6 stall codons set to $0.1s^{-1}$.
n_{stall}	6	N.U.	Number of stall codons.
$k_{preterm\_no\_hit\_intact}$, $k_{preterm\_5\_hit\_intact}$, $k_{preterm\_3\_hit\_intact}$, $k_{preterm\_both\_hit\_intact}$	0	s^{-1}	No abortive termination.
**Fig. 4B, Fig. 4C, SEC model**			
$k_{cleave\_no\_hit}$, $k_{cleave\_5\_hit}$	0.001	s^{-1}	Identical endonucleolytic mRNA cleavage rate at ribosomes that are not hit or hit only from mRNA exit side.
$k_{cleave\_3\_hit}$, $k_{cleave\_both\_hit}$	0	s^{-1}	No endonucleolytic mRNA cleavage at ribosomes that are hit from only mRNA entry side or both sides.
**Fig. 4B, Fig. 4C, CSEC model**			
$k_{cleave\_5\_hit}$, $k_{cleave\_both\_hit}$	0.001	s^{-1}	Identical endonucleolytic mRNA cleavage rate at ribosomes that are hit only from mRNA exit side or both sides.
$k_{cleave\_no\_hit}$, $k_{cleave\_3\_hit}$	0	s^{-1}	No endonucleolytic mRNA cleavage at ribosomes that are not hit or hit only from mRNA entry side.
**S3 Fig panel A**			**All parameters except ones below same as SEC model in Fig. 4B.**
$k_{cleave\_no\_hit}$, $k_{cleave\_5\_hit}$	0.0001, 0.002, 0.001	s^{-1}	Vary the endonucleolytic mRNA cleavage rate at ribosomes that are not hit or hit only from mRNA exit side.
**S3 Fig panel B**			**All parameters except ones below same as CSEC model in Fig. 4B.**
**S3 Fig panel B**, $n_{stall} = 1$			
x_{stall}	401	*codon*	Location of stall codons.
k_{elong_stall}	0.1	$codon/s^{-1}$	Elongation rate per codon in increased as n_{stall} in increased to keep total elongation rate across stall constant.
**S3 Fig panel B**, $n_{stall} = 2$			
x_{stall}	400:402	*codon*	Location of stall codons.

Continued on next page

Continued from previous page			
**Symbol**	**Value**	**Unit**	**Comment**
k_{elong_stall}	0.2	$codon/s^{-1}$	Elongation rate per codon in increased as n_{stall} in increased to keep total elongation rate across stall constant.
**S3 Fig panel B**, $n_{stall} = 3$			
x_{stall}	401:403	*codon*	Location of stall codons.
k_{elong_stall}	0.3	$codon/s^{-1}$	Elongation rate per codon in increased as n_{stall} in increased to keep total elongation rate across stall constant.
**S3 Fig panel B**, $n_{stall} = 4$			
x_{stall}	401:404	*codon*	Location of stall codons.
k_{elong_stall}	0.4	$codon/s^{-1}$	Elongation rate per codon in increased as n_{stall} in increased to keep total elongation rate across stall constant.
**S3 Fig panel B**, $n_{stall} = 5$			
x_{stall}	401:405	*codon*	Location of stall codons.
k_{elong_stall}	0.5	$codon/s^{-1}$	Elongation rate per codon in increased as n_{stall} in increased to keep total elongation rate across stall constant.
**S3 Fig panel B**, $n_{stall} = 6$			
x_{stall}	401:406	*codon*	Location of stall codons.
k_{elong_stall}	0.6	$codon/s^{-1}$	Elongation rate per codon in increased as n_{stall} in increased to keep total elongation rate across stall constant.
